# High Bioavailability of Bisphenol A from Sublingual Exposure

**DOI:** 10.1289/ehp.1206339

**Published:** 2013-06-12

**Authors:** Véronique Gayrard, Marlène Z. Lacroix, Séverine H. Collet, Catherine Viguié, Alain Bousquet-Melou, Pierre-Louis Toutain, Nicole Picard-Hagen

**Affiliations:** 1 INRA (Institut National de la Recherche Agronomique), UMR1331 (Unité Mixe de Recherche 1331), Toxalim, Research Center in Food Toxicology, Toulouse, France; 2 Université de Toulouse, INPT (Institut National Polytechnique de Toulouse), ENVT (Ecole Nationale Vétérinaire de Toulouse), EIP (Ecole d’Ingénieurs de Purpan), UPS (Université Paul Sabatier), Toulouse, France

**Keywords:** bioavailability, bisphenol A, endocrine disruptor, pharmacokinetic analysis, sublingual exposure

## Abstract

Background: Bisphenol A (BPA) risk assessment is currently hindered by the rejection of reported higher-than-expected plasma BPA concentrations in humans after oral ingestion. These are deemed incompatible with the almost complete hepatic first-pass metabolism of BPA into its inactive glucurono-conjugated form, BPA glucuronide (BPAG).

Objectives: Using dogs as a valid model, we compared plasma concentrations of BPA over a 24-hr period after intravenous, orogastric, and sublingual administration in order to establish the absolute bioavailability of BPA administered sublingually and to compare it with oral bioavailability.

Methods: Six dogs were sublingually administered BPA at 0.05 mg/kg and 5 mg/kg. We compared the time course of plasma BPA concentrations with that obtained in the same dogs after intravenous administration of the same BPA doses and after a 20-mg/kg BPA dose administrated by orogastric gavage.

Results: The data indicated that the systemic bioavailability of BPA deposited sublingually was high (70–90%) and that BPA transmucosal absorption from the oral cavity led to much higher BPA internal exposure than obtained for BPA absorption from the gastrointestinal tract. The concentration ratio of BPAG to BPA in plasma was approximately 100-fold lower following sublingual administration than after orogastric dosing, distinguishing the two pathways of absorption.

Conclusions: Our findings demonstrate that BPA can be efficiently and very rapidly absorbed through the oral mucosa after sublingual exposure. This efficient systemic entry route of BPA may lead to far higher BPA internal exposures than known for BPA absorption from the gastrointestinal tract.

## Introduction

Bisphenol A (BPA) is widely used in its monomeric form in the manufacture of polycarbonate plastics and epoxy resins [European Food Safety Authority (EFSA) 2006]. Vandenberg et al. (2007) suggested that the release of BPA monomers from consumer products leads to the contamination of drinking water, food, dust, and air, thus providing considerable potential for human exposure to BPA. In support of this suggestion are data reported by Calafat et al. (2008), who found measurable levels of BPA metabolites in > 90% of urine samples from a representative cohort of the U.S. population. The principal source of BPA exposure is through the diet and, based on the measurement of urinary concentrations of BPA metabolites as a biomarker of aggregate human exposure levels, the median exposure has been estimated at only 0.01–0.12 μg/kg/day [Food and Agriculture Organization of the United Nations/World Health Organization (FAO/WHO) 2010]. The current tolerable daily intake (TDI) is 0.05 mg/kg/day (EFSA 2006).

Widespread human exposure to BPA raises concern among regulatory agencies because of its estrogenic properties *in vitro* (Wetherill et al. 2007) and *in vivo* (Richter et al. 2007). The risk assessment for BPA is controversial because the TDI, which is based on guideline-driven toxicity studies (Ema et al. 2001; Tyl et al. 2002, 2008), is generally higher than doses that produce adverse effects on animals, especially if dosing occurs during the perinatal period (Cabaton et al. 2011; Vandenberg et al. 2008; vom Saal and Hughes 2005).

It is generally assumed that the undesirable effects of BPA are associated with plasma concentrations (internal dose) rather than the administered BPA dose. Thus, some researchers (Vandenberg et al. 2010) have questioned why reportedly high concentrations of unconjugated BPA in humans (in the nanograms per milliliter range) are not considered by regulatory agencies in the risk assessment process. Other researchers (Mielke and Gundert-Remy 2009) have noted that the relatively low estimated BPA daily intake and the observation of an extensive first-pass metabolism of oral BPA into its inactive glucurono-conjugated form (BPAG) (Völkel et al. 2002) are not consistent with those high plasma levels of BPA observed in biomonitoring studies.

Dekant and Völkel (2008) suggested that the high plasma BPA levels reported in humans may be due to artifacts related to sample preparation, storage, overestimation by analytical techniques, or background contamination from labware or indoor dust. However, there is no little or no direct evidence for this assertion, and there may be alternative explanations.

Because most BPA exposure in humans occurs via the mouth, we hypothesized that BPA could be bioavailable sublingually, which could contribute to higher plasma concentrations. In sublingual exposure, a substance diffuses into the blood through the mucous membrane under the tongue. The sublingual mucosa is highly vascularized; thus, a substance diffusing across this oral mucosal membrane has direct access to the systemic circulation via capillaries and venous drainage and will avoid first-pass hepatic metabolism (Patel et al. 2011).

In the present study, we used dogs to evaluate the oral transmucosal passage of BPA. The permeability of the buccal membrane is very similar in the dog and human, and thus the dog is a reliable species to assess sublingual absorption of drugs for human use (Barsuhn et al. 1988). The objectives of the study were *a*) to determine the bioavailability of BPA administered sublingually; *b*) to characterize the time course of the plasma BPA concentrations following sublingual BPA; and *c*) to compare systemic plasma BPA concentrations as a measure of exposure after sublingual and conventional oral dosing.

## Materials and Methods

*Animals*. Animals used in this study were treated humanely and with regard for the alleviation of suffering. All animal procedures were carried out in accordance with the accepted standards of humane animal care under agreement 31–242 for animal experimentation from the French Ministry of Agriculture. The study was conducted in six dogs (two male and four female Beagles; Harlan, Gannat, France). The dogs were 2–3 years of age and weighed 15–19 kg. They were housed in pairs in 12-m^2^ rooms, fed a standard diet, and had free access to drinking water. The animal rooms were illuminated by artificial light on a 12-hr light/dark cycle, and the temperature was maintained at about 20°C. The dogs had access to outdoor exercise areas for about 4 hr/day. Prior to the study day, the animals were fasted overnight and had free access to drinking water. They were given a standard meal 5 hr after dosing. During sampling periods, the dogs were housed individually in stainless steel cages.

*Test material and treatment*. BPA and all chemicals were purchased from Sigma-Aldrich (Saint-Quentin, Fallavier, France). For the 5-mg/kg BPA dose, solutions were prepared immediately before treatment by dissolving BPA (50 mg/mL) in 1% ethanol/49% propylene glycol (vol/vol) for the intravenous (iv) dose, ethanol for the sublingual bolus, or 40% ethanol/60% water (vol/vol) for sublingual drops. For the 0.05-mg/kg BPA dose, solutions were prepared immediately before treatment by dissolving BPA (0.5 mg/mL) in water containing 1% ethanol for iv and sublingual administration. For orogastric administration, BPA (40 mg/mL) was dissolved in 1% ethanol/9% corn oil (vol/vol).

*Experimental design and dosing*. Experiment 1. The first experiment was divided into two periods separated by 1 week. The dogs received BPA (5 mg/kg) by iv administration one period, and by sublingual administration during the other period using a crossover design. We chose the 5-mg/kg dose of BPA based on the iv dose we estimated to be required to achieve plasma BPA concentrations greater than the limit of quantification (LOQ; 1 ng/mL) for a period of about 8–10 hr (i.e., a duration sufficient to observe the terminal phase slope and allow calculation of BPA pharmacokinetic parameters). We used two different sublingual modalities of BPA administration: a single bolus and drops. A BPA solution (BPA at 5 mg/kg in ~ 1.3 mL ethanol) was deposited as a single bolus under the tongue of three dogs briefly anesthetized by an iv injection of sodium thiopental (Nesdonal, 11 mg/kg; Merial, Lyon, France). The three other dogs received the same volume and dose of BPA in the form of 20-µL drops of an aqueous solution containing 40% ethanol, which was continuously delivered toward the floor of the mouth over a 10-min period.

Experiment 2. In a second experiment, BPA was administered at a dose equivalent to the TDI (0.05 mg/kg/day), which was chosen in order to better reflect the maximal possible human BPA external exposure and to check the proportionality of BPA pharmacokinetics with dose. This experiment was divided into three periods, each separated by 1 week. During the first two periods, dogs were administered BPA (0.05 mg/kg) by iv and sublingual drop administration according to a crossover design as described above. During the third period, BPA (20 mg/kg) was administered by orogastric intubation. The 20-mg/kg BPA dose was selected based on pharmacokinetic data from a preliminary experiment in order to obtain plasma concentrations of unconjugated BPA of the same order as those observed after sublingual administration of BPA at the TDI dose level.

For both experiments, and for each dog, the iv BPA dose was administered as a bolus via an indwelling 22-G catheter under the same conditions of dose, volume, and anesthesia as during the corresponding sublingual administration.

*Blood sampling*. Serial jugular venous blood samples were collected before all administrations, and in the middle (5 min after commencement) and at the end of administration of sublingual drops. After sublingual drop administration ended and after iv and sublingual bolus administration, blood samples were collected at 2, 4, 8, 15, 30, 60, 90, 120, 180, and 240 min and every 2 hr for 12 hr (experiments 1 and 2) and at 24 hr for experiment 1. Serial blood samples were obtained at 15, 30, 60, 120, 180, and 240 min, every 2 hr for 12 hr, and at 24 hr after orogastric BPA administration (experiment 2).

Blood samples were collected into heparinized polypropylene tubes, immediately chilled on ice, and centrifuged for 10 min at 3,000 × *g* at 4°C. The supernatant plasma was stored in polypropylene tubes (Eppendorf) at –20°C until assay.

*BPA and BPAG assays*. BPA and BPAG in plasma samples were simultaneously quantified using an Acquity ultra performance liquid chromatograph coupled to a Xevo triple quadrupole mass spectrometer (both from Waters, Milford, MA, USA), according to a previously described method (Lacroix et al. 2011).

Briefly, samples (100 µL) were purified by protein precipitation, diluted with 150 µL acetonitrile and 50 µL of the internal standards deuterated BPA (BPA-d16) and ^13^C-labeled BPAG (BPAG ^13^C_12_), and separated on a C18 column with a water/acetonitrile gradient elution. The multiple reaction monitoring transitions used to detect BPA, BPA-d16, BPAG, and BPAG ^13^C_12_ were 227 > 212, 241 > 142, 403 > 227, and 415 > 239, with collision energies of 28, 20, and 30 eV, respectively. Chromatographic data were monitored by Targetlynx software (Waters). Blanks and quality control samples were used to monitor potential contamination during analysis and the accuracy and precision of the method.

The mean intra- and interday coefficients of variation for three concentration levels and for BPA and BPAG, respectively, were < 15%, and the LOQs were validated at 1 ng/mL and 5 ng/mL, respectively.

*Pharmacokinetic analysis*. Plasma concentration–​time profiles of BPA and BPAG were analyzed according to a noncompartmental approach using WinNonlin® Professional, version 5.3 (Pharsight Corporation, Cary, NC, USA). From the plasma BPA and BPAG concentration–time data in individual dogs, we derived the maximum concentration (C_max_) and the time to maximum concentration (T_max_). The areas under the BPA and BPAG plasma concentration–time curves (AUC_last_) and the areas under the first moment curves (AUMC_last_) were calculated using the linear trapezoidal rule from dosing time to the last sampling time. The mean residence time (MRT) was calculated as the ratio of AUMC_last_:AUC_last_.

In deriving the BPA pharmacokinetic parameters (AUC_last_, AUMC_last_, C_max_, T_max_, and MRT), we did not include the first 8 min immediately after sublingual administration ended because high plasma BPA levels encountered during this lag time may reflect the immediate input of BPA drained from the tongue into the jugular vein (the site of blood sampling) before it is diluted in the general circulation. The AUC_last_ values were normalized by the corresponding BPA dose.

For each dog, we determined the absolute bioavailability of BPA administered sublingually (both methods) at 5 mg/kg as the ratio of the normalized BPA AUC_last_ for the sublingual route to the equivalent AUC_last_ for the iv route. For the two BPA doses (0.05 and 5 mg/kg), we defined the extent of BPA sublingual absorption as the ratio of the BPAG AUC_last_ values obtained for the sublingual route to the equivalent BPAG AUC_last_ for the iv route. For 0.05 mg/kg BPA sublingual dosing, we considered this latter value to be an appropriate measure of the absolute bioavailability of BPA based on the assumption that BPAG is not formed at the site of administration or by a first pass effect. For computation of oral bioavailability and absorption rate, we consider only the iv dose of 5 mg/kg.

*Statistical analyses*. All results are presented as mean ± SD. Student’s *t*-test and SYSTAT12 software (Systat Software Inc., Chicago, IL, USA) were used to analyze differences in mean BPA and BPAG pharmacokinetic parameters (AUC_last_, C_max_, T_max_, and MRT) by route of administration.

## Results

BPA was not detected in any of the control samples obtained before BPA administration. The values for pharmacokinetic parameters of BPA and BPAG obtained for different doses via different routes (iv, sublingual, and orogastric) of BPA administration are presented in Tables 1 and 2. As noted above, calculations of pharmacokinetic parameters omitted the BPA plasma concentrations obtained during and up to 8 min after the end of sublingual administration of BPA.

**Table 1:** Mean (± SD) values for pharmacokinetic parameters of BPA after iv, sublingual, and orogastric BPA dosing.

Pharmacokinetic parameter^*a*^	iv		Sublingual		Orogastric
	0.05mg/kg	5mg/kg	0.05mg/kg	5mg/kg	20mg/kg
C_max_ (ng/mL)	64±36	7,296±1,615	249±331	6,443±3,910	47±20
T_max_ (min)	2±0	3±1	10±4	13±9	20±8
AUC_last_ (× 10^3^ng/min/mL)	1±0	221±54	2±1	145±44*	6±2
MRT (min)	NC	69±13	NC	73±33	112±37
BPA bioavailability (%)	NA	NA	NC	70±31	0.72±0.28
Abbreviations: NA, not applicable; NC, not calculated. ^***a***^The first 8 min following the end of sublingual adminis­tration were not taken into account in deriving the BPA pharmaco­kinetic parameters. *p ≤0.05, compared with iv administration of the same BPA dose by Student’s *t*-test.					

**Table 2:** Mean (± SD) values for pharmacokinetic parameters of BPAG after iv, sublingual, and orogastric BPA dosing.

Pharmacokinetic parameter	iv		Sublingual		Orogastric
	0.05mg/kg	5mg/kg	0.05mg/kg	5mg/kg	20mg/kg
C_max_ (ng/mL)	78±38	15,657±6,426	46±20*	11,808±10,419	30,777±13,604
T_max_ (min)	12±4	16±7	35±20	35±13*	38±18
AUC_last_ (× 10^3^ng/min/mL)	8±5	2,884±776	7±5	2,355±893	6,081±1,935
MRT (min)	NC	417±65	NC	562±164	501±200
BPA absorption and/or bioavailability (%)	NA	NA	90±26	81±18	54±19
Abbreviations: NA, not applicable; NC, not calculated. *p ≤0.05, compared with iv administration of the same BPA dose by Student’s *t*-test.					

**Figure 1:**
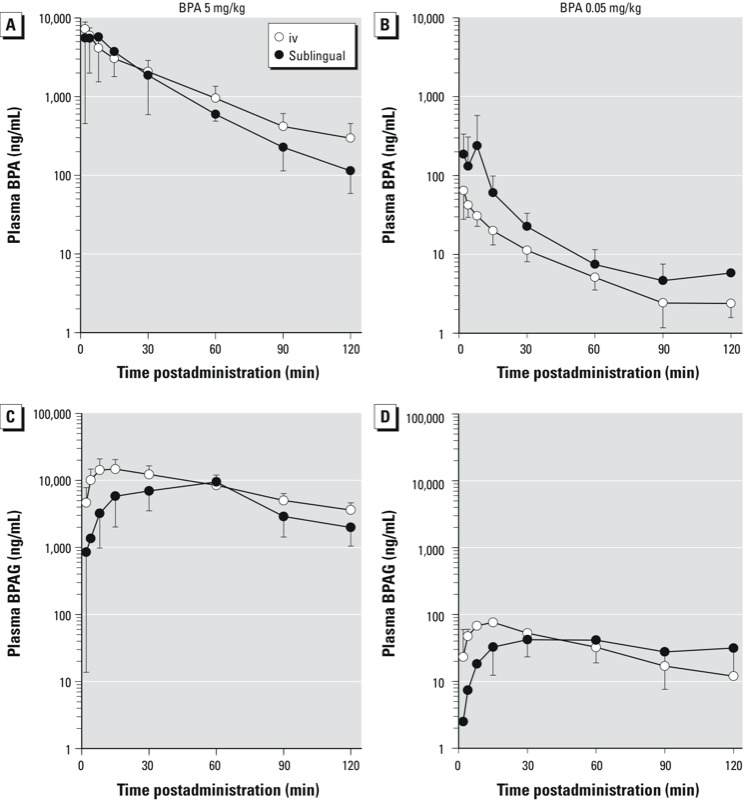
Semilogarithmic plots of mean (± SD) plasma concentrations (ng/mL) of BPA (*A,B*) and BPAG (*C,D*) versus time after a single iv (*n* = 6) or sublingual (*n* = 6) administration of BPA at 5 mg/kg (*A,C*) and 0.05 mg/kg (*B,D*). Time 0 represents the end of BPA administration.

*Experiment 1: iv and sublingual dosing (BPA 5 mg/kg)*. Because the two sublingual administration methods (bolus and drops) gave comparable results, they were combined into one data set. The time course of mean plasma BPA concentrations after sublingual BPA administration was very similar to that obtained in the same dogs after iv administration (Figure 1A); however, the plasma concentrations during the first minutes after sublingual application were higher than those obtained after iv administration.

For the 5-mg/kg BPA sublingual dose, the mean BPA T_max_ was 13 ± 9 min (mean ± SD) (Figure 1A, Table 1). The mean BPA C_max_ for this sublingual dose was not significantly different from the corresponding value obtained after iv administration (7,296 ± 1,615 ng/mL for iv vs. 6,443 ± 3,910 ng/mL for sublingual; *p* = 0.6; Table 1). The BPA MRT was not significantly different according to the iv versus sublingual routes of administration (69 ± 13 vs. 73 ± 33 min; *p* = 0.7; Table 1).

For BPAG, the mean C_max_ did not significantly differ by route of administration (15,657 ± 6,426 vs. 11,808 ± 10,419 ng/mL for iv vs. sublingual, respectively; *p* = 0.2, Table 2). However, the BPAG T_max_ was delayed (Figure 1C, Table 2) compared with the iv route (16 ± 7 vs. 35 ± 13 min for iv vs. sublingual; *p* = 0.04).

**Figure 2:**
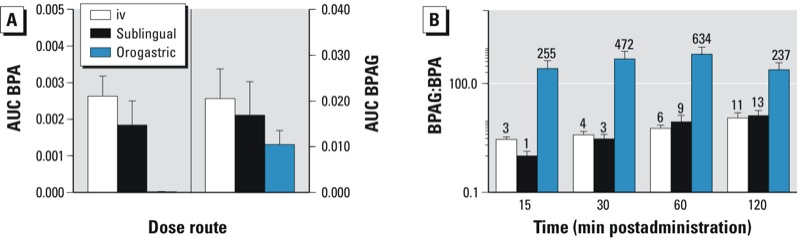
Mean (± SD) BPA AUC_last_ and BPAG AUC_last_ normalized for the actual administered dose (*A*) and semilogarithmic plot of the mean ratio of BPAG:BPA molar concentrations versus time (*B*). The numbers above the bars represent the mean value of the BPAG:BPA molar concentrations.

The mean BPA AUC_last_ (normalized to administered dose) in response to sublingual administration of BPA at 5 mg/kg was lower than that obtained after iv administration (*p* = 0.04; Table 1, Figure 2A), whereas the corresponding mean BPAG AUC_last_ values were not significantly different. The mean BPA bioavailability for the high sublingual dose was 70 ± 31%. This high systemic bioavailability was confirmed by the mean ratio of BPAG AUC values (81 ± 18% for sublingual dosing), which is also an estimate of the systemic bioavailability, provided that the BPAG is not formed at the administration site or by a first-pass effect, which seems to be a reasonable assumption for a direct buccal absorption.

*Experiment 2: iv and sublingual dosing (BPA 0.05 mg/kg)*. BPA was not detected by about 2 hr after iv or sublingual BPA administration (0.05 mg/kg), whereas BPAG plasma levels in three dogs remained > LOQ for 8–10 hr after BPA administration.

Following sublingual applications of BPA at 0.05 mg/kg, the BPA plasma levels were more variable and higher than those obtained in the same dogs after iv administration of the same dose (Figure 1B).

The mean T_max_, not counting the 8 min immediately after sublingual BPA administration (0.05 mg/kg) ended, was 10 ± 4 min (Figure 1B, Table 1). The mean BPA C_max_ after sublingual administration was more variable than the corresponding value obtained after iv administration (64 ± 36 ng/mL vs. 249 ± 331 ng/mL, for iv vs. sublingual administration; Table 1). For BPAG, the mean C_max_ after sublingual dosing was lower than that after iv administration (78 ± 38 for iv vs. 46 ± 20 ng/mL for sublingual; *p* = 0.03; Table 2). However, the BPAG T_max_ after sublingual administration was delayed (35 ± 20 min; Figure 1D, Table 2) compared with the iv route (12 ± 4 min; *p* = 0.06).

As reflected by the mean BPA AUC_last_, the systemic BPA exposure resulting from sublingual dosing of BPA at 0.05 mg/kg was more variable and higher than that obtained after iv administration and was thus not considered for bioavailability computation.

The mean BPA sublingual bioavailability for the 0.05 mg/kg dose was 90 ± 26%, as computed by the mean ratio of the BPAG AUC_last_ values obtained for the sublingual route to the equivalent BPAG AUC_last_ for the iv route.

*Orogastric BPA dosing (20 mg/kg)*. The mean C_max_ and T_max_ values of plasma BPA observed after orogastric administration of BPA at 20 mg/kg were 47 ± 20 ng/mL and 20 ± 8 min, respectively. The mean BPA bioavailability was 0.72 ± 0.28%. This value was lower than the mean ratio of BPAG AUC values (54 ± 19%), showing that BPA was rather well absorbed by the gastrointestinal tract but that most absorbed BPA is metabolized by a first-pass effect at the hepatic level. A major difference between the two modalities of oral administration (orogastric vs. sublingual) was the BPAG:BPA plasma molar concentration ratio. During the first 120 min after sublingual administration of BPA at 5 mg/kg, the mean BPAG:BPA ratio ranged from 1:1 to 13:1. During the 120 min that followed sublingual dosing with BPA at 0.05 mg/kg, this ratio ranged between 1:1 and 6:1. This was almost 100 times lower than the value obtained after BPA absorption from the gastrointestinal tract after orogastric dosing, which ranged from 237:1 to 634:1 over the same time period (Figure 2B).

## Discussion

Much of the concern regarding BPA safety in humans has centered on the adverse effects of BPA in experimental animal studies, in which blood concentrations of BPA were close to those of unconjugated BPA (in the nanograms-per-milliliter range) that have been reported in numerous human biomonitoring surveys (Vandenberg et al. 2010). However, these high BPA concentrations are considered to be erroneous and have been discounted for risk assessment purposes *a*) because of their deemed incompatibility with the low BPA estimated daily intake, which is mainly through the diet (FAO/WHO 2010); and *b*) because of the tenet based on oral pharmacokinetic data in humans, which indicate extensive hepatic first-pass glucuronidation of BPA leading to inactivation of almost all ingested BPA (Völkel et al. 2002).

To our knowledge, the present study is the first to demonstrate that BPA delivered sublingually is almost totally bioavailable. Indeed, this pathway of BPA absorption allows hepatic first-pass glucuronidation to be bypassed, leading to much higher internal BPA exposures than those obtained after conventional oral administration.

In our study we used an *in vivo* dog model to establish the systemic uptake of sublingually administered BPA. The relevance of this model is supported by similarity of the mechanisms of drug transport and of histology of the dog buccal mucosa compared with human buccal mucosa (Barsuhn et al. 1988), which is not the case for rats where the buccal epithelium is keratinized (Shojaei 1998). In the present study, we selected the jugular vein as the site of blood sampling because it allows the collection of blood from the venous drainage of the tongue. Thus, the fact that jugular blood BPA concentrations were transiently higher after sublingual administration than were those obtained after iv administration of the same dose suggests a rapid and efficient passage of BPA by the transmucosal oral route. The disadvantage of this blood collection site is that the corresponding plasma BPA concentrations do not properly reflect systemic exposure during the sublingual absorption phase (Sohlberg et al. 2013), that is, before mixing of the jugular blood with systemic blood. For this reason, in the evaluation of the systemic exposure to BPA and in deriving the BPA AUC values for BPA sublingual dosing, in the kinetic analysis we discounted the plasma BPA concentrations measured in jugular blood during the BPA administration itself and during the 8-min immediately after the sublingual application ended. We considered this delay sufficient to not bias the bioavailability estimation because the BPA MRT values did not differ between the iv and sublingual routes, indicating a very short buccal (sublingual) absorption phase of about a few minutes. In a separate experiment performed on two of the six dogs used in this study, we collected blood samples in parallel from the jugular and the cephalic veins after BPA sublingual dosing at both 0.05 and 0.5 mg/kg BPA doses. We observed that after sublingual dosing with BPA at these doses, plasma BPA concentrations in the jugular vein were higher and more variable than corresponding concentrations in the cephalic vein during the first 60 min and 15 min after dosing, respectively [see Supplemental Material, Figure S1 (http://dx.doi.org/10.1289/ehp.1206339)]. Systemic BPA exposures estimated from blood samples taken from the cephalic vein represented about 57% and 94% of that estimated from jugular blood samples obtained from 8 min following the completion of BPA sublingual dosing at 0.05 and 0.5 mg/kg, respectively (see Supplemental Material, Table S1); this result indicates that for the 5-mg/kg BPA dose, the BPA bioavailability (70%) was properly calculated from jugular blood BPA concentrations obtained from the 8 min following the completion of BPA administration. This finding was supported by the high extent of BPA bioavailability computed using systemic exposure to BPAG (81%). Indeed, bioavailability can be also determined by the AUC ratio of the metabolite, provided that the metabolite is not formed at the site of administration or by a first-pass effect (Cutler 1981; Weiss 1990); that seems to be a reasonable assumption for a direct buccal (sublingual) absorption.

The mean absolute BPA bioavailability resulting from sublingual administration (70%) computed using plasma BPA concentrations after the administration of BPA at 5 mg/kg showed high bioavailability. For this experiment, we used an ethanol vehicle (40–100% ethanol) and a highly concentrated dosing solution for sublingual administration; thus, a vehicle effect facilitating the sublingual absorption cannot be ruled out. To check the relevance of our findings with the 5-mg/kg BPA dose, in our second experiment, we administered BPA in an aqueous solution containing 1% ethanol and a 100-times lower BPA dosage, corresponding to the TDI (0.05 mg/kg). In that experiment, the fact that BPA was no longer detected about 2 hr after iv and sublingual BPA administration prevented accurate evaluation of the terminal slope and of two BPA pharmacokinetic parameters (AUC_last_ and bioavailability) that were more accurately evaluated after the administration of the 5-mg/kg dose. However, the systemic exposure observed after sublingual dosing of BPA at 0.05 mg/kg, compared with that obtained after iv administration of the same BPA dose, clearly indicates that the findings obtained for the high BPA dose are consistent with those obtained with a lower dose level. In addition when considering BPAG, the bioavailability of BPA after administration of a low BPA dose (0.05 mg/kg) can be properly computed and was 90%.

The physicochemical properties of BPA, namely, its moderate water solubility (LogP of 3.3), and its relative low molecular weight (228), are likely to explain its penetration across the sublingual membrane and may explain the high extent of BPA absorption.

The use of this *in vivo* canine model showed that the extensive uptake of BPA following sublingual applications, which bypasses the hepatic first-pass glucuronidation mechanism, may lead to a internal BPA exposure about 100-fold higher than that obtained after orogastric administration of the same external BPA dose. The markedly increased internal BPA exposure resulting from transmucosal absorption highlights the possible limitations of those studies in which BPA was administered as a single oral bolus (Doerge et al. 2010a, 2010b). These limitations were discussed by Sieli et al. (2011), who reported some differences in BPA internal exposures in mice after exposure through the diet versus a single oral bolus. The results of the present study suggest that the presence of BPA in food may increase the internal exposure to bioactive BPA in animals and humans compared with a single bolus oral administration, although in rodents the totally keratinized oral mucosal lining (Shojaei 1998) may limit transmucosal BPA absorption. Currently, the results of Teeguarden et al. (2011) do not support sublingual absorption as a major contributor of dietary BPA to a much higher than expected human internal exposure. The conditions controlling absorption after sublingual dosing in our experimental design may be different from those prevailing during oral exposure to BPA contained in food or dust. The potential contribution of sublingual absorption of BPA to high blood unconjugated concentrations (in the nanogram-per-milliliter range) must be evaluated through biomonitoring surveys designed to integrate both dietary and nondietary sources of BPA, including the potential nondietary ingestion route associated with hand-to-mouth activity. Indeed, a meta-analysis addressing the question of mouthing behaviors in children have shown that the frequency of hand-to-mouth activity (i.e., up to 28 contacts per hour) is an important variable for exposure assessments (Xue et al. 2007). Considering the potential nonfood sources of BPA, it is important to note that a significant amount of BPA can be released from resin-based dental materials, estimated at 13 µg and 30 mg of BPA in the average and the worst case scenarios, respectively, after one full crown restoration of a molar (Geens et al. 2012; Van Landuyt et al. 2011) and that BPA present in thermal papers may be taken in orally through direct contact of unwashed hands with the mouth (Geens et al. 2012).

Another major finding of the present study is that the two pathways of BPA systemic availability (i.e., with or without a hepatic first-pass effect) can be easily distinguished by taking into account the plasma BPAG:BPA molar concentration ratio. After BPA entry into the systemic circulation by the sublingual route, this ratio was about 100 times lower than that obtained after orogastric administration. This ratio obtained after orogastric dosing in dogs is consistent with data on oral pharmacokinetics in humans and nonhuman primates that showed that the peak serum concentrations of unconjugated BPA after oral administration are approximately 0.2–1% (Völkel et al. 2002) and 0.1–3% of the total (unconjugated plus conjugated) BPA (Doerge et al. 2010b; Taylor et al. 2011; Tominaga et al. 2006). The remarkably lower BPAG:BPA ratio obtained after sublingual administration justifies the claim of differences relating to systemic absorption bypassing hepatic metabolizing enzymes. These data suggest that unconjugated BPA concentrations in human serum associated with a BPAG:BPA plasma concentration ratio of < 10 are achievable. It follows that such data do not have to be attributed to sample contamination. Therefore, recent data indicating that BPAG is not abundant in human serum relative to total BPA levels (Kosarac et al. 2012) should be reevaluated in light of the present results demonstrating a possible direct systemic entry of BPA from sublingual absorption.

## Conclusions

The finding that BPA can be efficiently and very rapidly absorbed by the sublingual route suggests that that sublingual absorption of BPA that enters the mouth from both dietary and nondietary sources may contribute to much higher internal exposure to the unconjugated form of BPA than would be expected after the passage through the gastrointestinal tract. Our study further shows that the BPAG:BPA plasma concentration ratio clearly differentiates the routes of BPA entry into to the systemic circulation with or without bypassing the liver. This finding is likely to have major implications for the interpretation of human biomonitoring data; such interpretation should take into account that BPA concentrations in blood cannot directly be extrapolated from the BPAG levels by assuming a systematic extensive hepatic first-pass effect under all circumstances.

## Supplemental Material

(324 KB) PDF
