# Supplementary material for: High Bioavailability of Bisphenol A from Sublingual Exposure
Source: Environ Health Perspect. 2013 Jun 12;121(8):951–6. doi: 10.1289/ehp.1206339 (PMC3734497; doi:10.1289/ehp.1206339)
Supplement: (324 KB) PDF [file ehp.1206339.s001.pdf]

SUPPLEMENTAL MATERIAL

High Bioavailability of Bisphenol A from Sublingual Exposure

Véronique Gayrard[1,2], Marlene Z. Lacroix[1,2], Severine H. Collet[1,2], Catherine Viguié[1,2], Alain Bousquet-Melou[1,2], Pierre-Louis Toutain[1,2], Nicole Picard-Hagen[1,2]

[1] INRA (Institut National de la Recherche Agronomique), UMR1331 (Unité Mixe de Recherche 1331), Toxalim, Research Center in Food Toxicology, F-31027 Toulouse, France

[2] Université de Toulouse, INPT (Institut National Polytechnique de Toulouse), ENVT (Ecole Nationale Vétérinaire de Toulouse), EIP (Ecole des Ingénieurs de Purpan), UPS (Université Paul Sabatier), F-31076 Toulouse, France

Corresponding author: Pierre-Louis Toutain

UMR1331 Toxalim

Ecole Nationale Vétérinaire de Toulouse, Laboratoire de Physiologie

23 chemin des Capelles, BP 87614

31076 Toulouse cedex 3

France

Phone : (33) 561 193 915

Email: pl.toutain@envt.fr

Table of contents

Supplemental Material, Experimental Design and Methods Page 2

Supplemental Material, Figure S1 Page 3

Supplemental Material, Tables S1 and S2 Page 4

Supplemental Material, Experimental Design and Methods

In a supplementary experiment, two of the dogs previously used received sublingual administrations of BPA at 2 doses, 0.05 and 0.5mg/kg, one week apart. BPA was sublingually delivered as repeated deposits of 20 µl drops of an aqueous solution containing 1% ethanol as described in the main experiment. Blood samples were collected both from the leg (cephalic vein) and the jugular vein before and at 2, 4, 8, 15, 30, 45, 60, 90, 120, 180, 240 min and every 2-h for 10 h after BPA sublingual BPA dosing. BPA and BPA-G in plasma samples were quantified and pharmacokinetic analyses were performed as previously described.

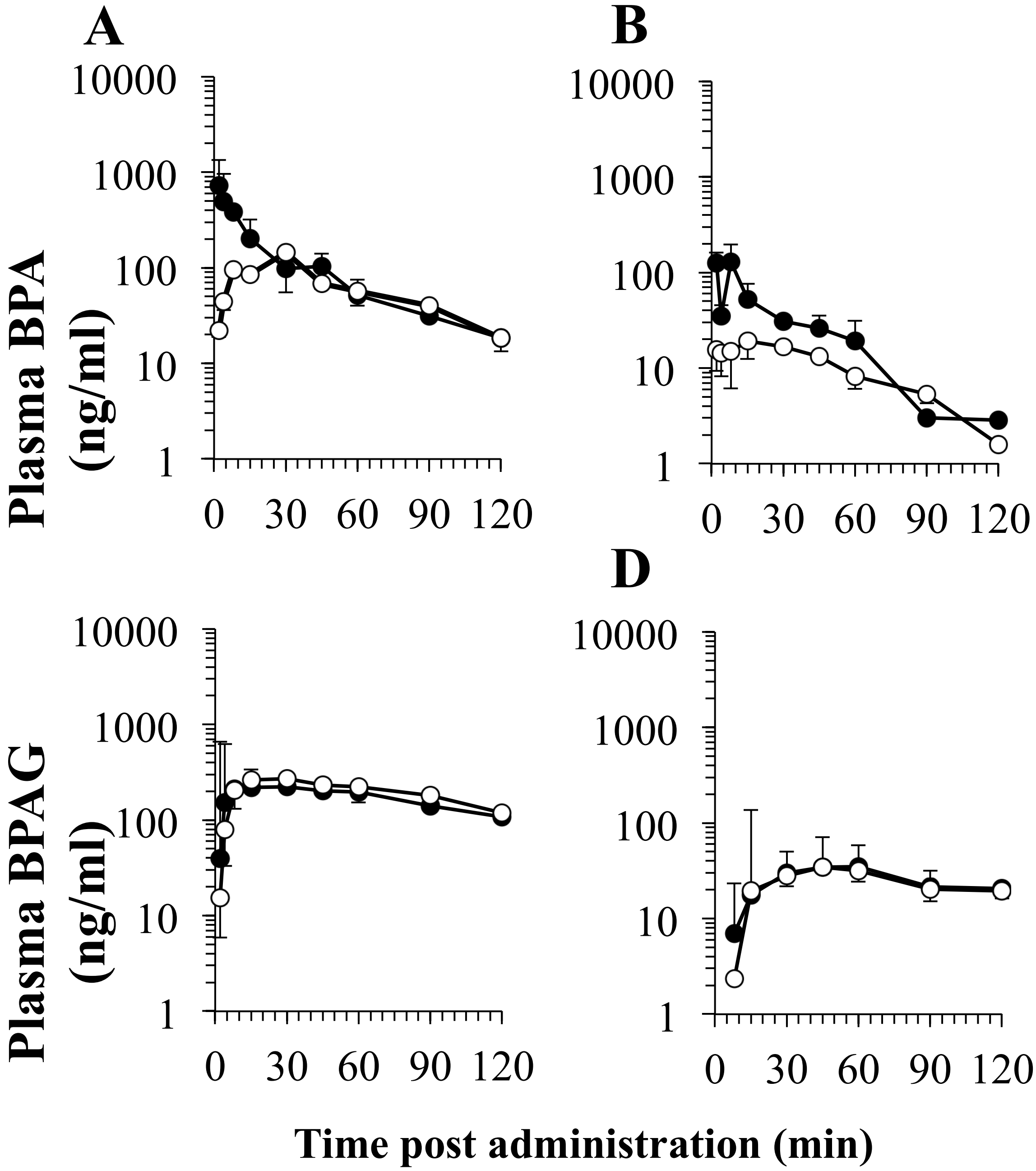

Supplemental Material, Figure S1: Semi-logarithmic plots of mean (± SD) plasma concentrations (ng/mL) of BPA and BPAG *versus* time (min) after BPA dosing at 0.5mg/kg (A, C) and 0.05 mg/kg (B, D), respectively. Blood samples were collected both from the leg vein (cephalic vein, open symbols, n=2) and the jugular vein (closed symbols, n=2). Time 0 represents the end of the administrations.

Supplemental Material, Table S1: Mean values (±SD) for pharmacokinetic parameters of BPA following sublingual BPA dosing at 0.05 and 0.5mg/kg. Blood samples were collected both from the leg vein (cephalic vein, n=2) and the jugular vein (n=2).

Pharmacokinetic parameter[a]	0.05mg/kg Jugular vein	0.05mg/kg Cephalic vein	0.5mg/kg Jugular vein	0.5mg/kg Cephalic vein
Cmax (ng/ml)	130 ± 68	20 ± 6	384 ± 16	151 ± 81
Tmax (min)	8 ± 0	23 ± 11	8 ± 0	23 ± 11
AUClast (ng.min/ml)	2366 ± 980	1186 ± 180	10056 ± 3163	9524 ± 3566

[a]The first 8 min following the end of sublingual administrations were not taken into account to derive the BPA pharmacokinetic parameters from jugular plasma BPA concentrations

Supplemental Material, Table S2: Mean values (±SD) for pharmacokinetic parameters of BPAG following sublingual BPA dosing at 0.05 and 0.5mg/kg. Blood samples were collected both from the leg vein (cephalic vein, n=2) and the jugular vein (n=2).

Pharmacokinetic parameter	0.05mg/kg Jugular vein	0.05mg/kg Cephalic vein	0.5mg/kg Jugular vein	0.5mg/kg Cephalic vein
Cmax (ng/ml)	43 ± 6	36 ± 1	266 ± 57	292 ± 4
Tmax (min)	53 ± 11	53 ± 11	19 ± 16	23 ± 11
AUClast (ng.min/ml)	3658 ± 465	3283 ± 453	36632 ± 8530	45835 ± 9390
